# Nuclear division cycle 80 promotes malignant progression and predicts clinical outcome in colorectal cancer

**DOI:** 10.1002/cam4.1284

**Published:** 2018-01-17

**Authors:** Xuebing Yan, Linsheng Huang, Liguo Liu, Huanlong Qin, Zhenshun Song

**Affiliations:** ^1^ Department of General Surgery Shanghai Tenth People's Hospital Tongji University School of Medicine No. 301, Yan‐chang Road Shanghai 200072 China; ^2^ Anhui Medical University No. 81, Mei‐shan Road Hefei 230032 China; ^3^ Department of General Surgery Shanghai Jiao Tong University Affiliated Sixth People's Hospital No. 600, Yi‐shan Road Shanghai 200233 China

**Keywords:** Biomarker, colorectal cancer, DUSP5, FOXO1, NDC80

## Abstract

Colorectal cancer (CRC) is a common human malignancy worldwide and increasing studies have attributed its malignant progression to abnormal molecular changes in cancer cells. Nuclear division cycle 80 (NDC80) is a newly discovered oncoprotein that regulates cell proliferation and cycle in numerous malignancies. However, its clinical significance and biological role in CRC remain unclear. Therefore, in this study, we firstly analyze its expression in a retrospective cohort enrolling 224 CRC patients and find its overexpression is significantly correlated with advanced tumor stage and poor prognosis in CRC patients. In addition, our result reveals it is an independent adverse prognostic factor affecting CRC‐specific and disease‐free survival. The subgroup analysis indicates NDC80 expression can stratify the clinical outcome in stage II and III patients, but fails in stage I and IV patients. In cellular assays, we find knockdown of NDC80 dramatically inhibits the proliferative ability, apoptosis resistance, cell cycle progression, and clone formation of CRC cells in vitro. Using xenograft model, we further prove knockdown of NDC80 also inhibits the tumorigenic ability of CRC cells in vivo. Finally, the microarray analysis is utilized to preliminarily clarify the oncogenic molecular mechanisms regulated by NDC80 and the results suggest it may promote CRC progression partly by downregulating tumor suppressors such as dual specificity phosphatase 5 and Forkhead box O1. Taken together, our study provides novel evidences to support that NDC80 is not only a promising clinical biomarker but also a potential therapeutical target for CRC precise medicine.

## Introduction

Colorectal cancer (CRC) is the third most common human malignancy in the United States, accounting for an estimated 135,430 newly diagnosed cases and 50,260 cancer‐related deaths in 2017 1. Although increased screening and improved treatment have been found to effectively reduce CRC mortality during the past decade, significant prognostic disparities still exist in patients within the same pathological stage 2. This challenging situation may largely reflect the shortage of reliable biomarkers used for precise diagnosis and treatment. Recent studies have strongly supported the clinical applicability of molecular classification in characterizing CRC patients, such as microsatellite instability, chromosome instability, and deficient mismatch repair 3. However, these encouraging achievements are far from sufficient to improve patient survival because CRC development is a multistep process involving complicated molecular changes and our knowledge of this field is relatively limited. Therefore, tremendous efforts are still essential for further clarifying CRC‐related molecular events and identifying more promising molecular biomarkers used for clinical management.

Cell cycle alteration is a key molecular event contributing to tumor development and targeting the involved molecules may be beneficial for anticancer therapies 4. Mitosis is a crucial phase during cell cycle, where tumor cell death could be induced by triggering spindle assembly checkpoint (SAC) 5. This point strongly promotes us to investigate whether mitotic proteins have the capacity to be useful therapeutical targets or clinical indicators for CRC patients. Nuclear division cycle 80 (NDC80), also called as highly expressed in cancer 1 (HEC1), is a newly discovered mitotic protein that regulates cell cycle through interacting with SAC protein kinase 6, 7. It is also found to drive mitotic progression through binding with phosphorylated spindle and kinetochore‐associated protein 3 8. Recently, emerging studies have demonstrated that NDC80 is abnormally expressed in human malignancies and may play a crucial role in carcinogenesis. For example, NDC80 is overexpressed in prostate cancer patients and promotes cancer cell growth in vitro via regulating long noncoding RNA BX647187 9. Meng et al. have proved NDC80 overexpression is correlated with advanced tumor stage in pancreatic cancer patients, and its downregulation inhibits the proliferative and antiapoptotic ability of cancer cells 10. In addition, our previous work has demonstrated NDC80 expression can stratify the clinical outcome in osteosarcoma patients, implying its potential to be a prognostic predictor 11. Taken together, these evidences collectively suggest that NDC80 may be a promising molecular biomarker for cancer diagnosis and treatment.

However, despite numerous studies regarding NDC80, its specific biological role in CRC remains unclear. Therefore, in this study, we first determined the expression and clinical significance of NDC80 in a retrospective cohort enrolling 224 CRC patients. Second, cellular assays and xenograft model were used to investigate the oncogenic role of NDC80 in vitro and in vivo. Finally, microarray and ingenuity pathway analysis was utilized to preliminarily clarify the underlying molecular mechanisms regulated by NDC80 in CRC development.

## Materials and Methods

### Patient data and tissue specimens

In this study, pairs of tumor tissues and matched adjacent normal tissues were collected from 224 CRC patients who received surgical treatment at Department of General surgery, Shanghai Tenth People's Hospital, Tongji University School of Medicine, between November 2008 and January 2016. All the patients were pathologically diagnosed as CRC and none of them received preoperative chemoradiotherapy. Postoperative tumor‐node‐metastasis (TNM) stage was determined according to Union for International Cancer Control (UICC) staging system (8th edition). For stage II patients with high‐risk factors and stage III/IV patients, a standard chemotherapy scheme (FOLFOX) was performed on well‐tolerated individuals. The follow‐up surveillance was performed regularly including CEA detection, physical, and radiological examination every 3 months for the first 2 years and every 6 months for the remaining 3 years. The patient prognosis was assessed using CRC‐specific survival (CSS) and disease‐free survival (DFS). CSS is defined as the time interval from surgery to CRC‐related death, whereas DFS is defined as the time interval from surgery to local recurrence or lymph node/distant metastasis. This study was approved by the ethics committee of Shanghai Tenth People's Hospital, Tongji University School of Medicine. Written informed consents were obtained from patients for using their clinical data and surgical specimens in noncommercial scientific researches.

### Quantitative real‐time polymerase chain reaction

Total RNA was extracted from fresh tissues or cultured cells using Trizol Kit (Thermo Fisher Scientific, USA) according to the manufacturer's instructions. The obtained RNA was reverse‐transcribed to synthesize first‐strand cDNA using M‐MLV Reverse Transcriptase (Promega, USA). Then, the polymerase chain reaction was performed on a 7500 Real‐Time PCR System (Applied Biosystems, USA) using SYBR^®^ Premix Ex TaqTM kit (Takara, Japan). The cycling condition for PCR was applied as follows: 7 sec for melting at 95°C, 10 sec for annealing at 57°C, and 15 sec for extending at 72°C. The primer sequences of genes were provided in Table S1. The relative mRNA level of each gene was calculated using the 2^−ΔΔ*T*^ method and GAPDH was utilized as an internal control.

### Western blot

Total protein was extracted from fresh tissues or cultured cells using lysis buffer (Genechem, China) and the protein concentration was detected using the bicinchonininc acid (BCA) method. The protein samples were then subjected to 10% sodium dodecyl sulfate polyacrylamide gel electrophoresis (SDS‐PAGE) and transferred onto polyvinylidene difloride (PVDF) membranes. The nonspecific binding was blocked using skim milk and the membranes were incubated with the following primary antibodies overnight at 4°C: anti‐NDC80 (1:1000, Abcam, UK), anti‐Dual specificity phosphatase 1 (DUSP1) (1:1000, Abcam), anti‐Dual specificity phosphatase 5 (DUSP5) (1:1000, Abcam), anti‐Forkhead box O1 (FOXO1) (1:1000, Cell Signaling Technology, USA), and anti‐GAPDH (1:2000, Santa Cruz Biotechnology, USA). After three washes with PBS solution, the membranes were incubated with Horseradish Peroxidase‐conjugated secondary antibody (1:5000, Santa Cruz Biotechnology) for 1 h at room temperature. Finally, protein expression was visualized using Chemiluminescence Detection Kit (Thermo Fisher Scientific) and GAPDH serves as an internal control.

### Immunohistochemistry and staining evaluation

Immunohistochemical staining was performed according to our previous description 12. In brief, tissue samples were fixed in methanol, embedded in paraffin and cut into 4‐*μ*m‐thick sections. The sections were then dewaxed in xylene, rehydrated in gradient alcohol, and subjected to microwave heating for antigen retrieval. Endogenous peroxidase activity was subsequently blocked by incubation with 3% hydrogen peroxide solution for 5 min. After three washes with PBS solution, the sections were successively incubated with the primary antibody against NDC80 (1:500) overnight at 4°C and secondary antibody (1:250, Abcam) for 30 min at 37°C. The protein staining was visualized using Diaminobenzidine Kit (Thermo Fisher Scientific). Finally, the sections were counterstained with hematoxylin and sealed with neutral balsam. Negative controls were prepared by incubating the sections with antibody diluent instead of primary antibodies.

Staining evaluation was performed by two researchers who are blind to patient data. The evaluation criteria were based on scores of Staining Intensity (SI) and Percentage of Positive area (PP). SI was classified as follows: score 0 (negative), score 1 (weak), score 2 (moderate), and score 3 (strong). PP was classified as follows: score 0 (0–5%), score 1 (6–25%), score 2 (26–50%), score 3 (51–75%), and score 4 (76–100%). A final score was calculated by multiplying both the scores and then analyzed in a receiver operating characteristic (ROC) curve for determining its cut‐off value. A final score more or less than its cut‐off value indicates high or low expression, respectively.

### Cell culture and RNA interference

Human CRC cell lines (LoVo, HCT‐116, SW620, SW480, Caco‐2, and HT‐29) were purchased from the Type Culture Collection of the Chinese Academy of Sciences (Shanghai, China). The culture mediums were supplemented with 10% fetal bovine serum and 1% penicillin/streptomycin (Thermo Fisher Scientific), and prepared for specific cell lines as follows: McCoy's 5A culture medium (Sigma‐Aldrich, USA) for HCT‐116 and HT‐29 cell line, L‐15 culture medium (Thermo Fisher Scientific) for SW620 and SW480 cell line, F12K (Sigma‐Aldrich), and MEM (Thermo Fisher Scientific) culture medium for LoVo and Caco‐2 cell line, respectively. All the cell lines were cultured in a humidified atmosphere containing 5% CO_2_ at 37°C.

Short hairpin RNA (shRNA) was utilized to stably downregulate NDC80 expression in CRC cells. The sequences of shRNA and its negative control (NC) are designed as follows: shRNA:5′‐CATTCTTGACCAGAAATTA‐3′; NC:5′‐TTCTCCGAACGTGTCACGT‐3′. The lentivirus product was purchased from Genechem and transfection procedure was performed in 293T cells using Lipofectamine 2000 (Thermo Fisher Scientific) as described previously 13. Then, CRC cells in logarithmic phase were infected with lentivirus carrying shRNA or NC based on multiplicity of infection (MOI). The interference efficacy of shRNA was examined by Quantitative real‐time polymerase chain reaction (qRT‐PCR) and western blot.

### Cell Counting Kit‐8 assay

CRC cells in logarithmic phase were seeded into a 96‐well plate with 2000 cells per well. After overnight incubation, each well was supplemented with 10 *μ*L of Cell Counting Kit‐8 (CCK‐8) reagent (Sigma‐Aldrich). After 4 h incubation, the plate was transferred onto a microplate reader (Tecan, Switzerland) for detecting the optical density (OD) value of each well at 450 nm.

### Cell apoptosis and cycle assay

The apoptosis rate and cycle distribution of CRC cells were detected by flow cytometry (Millipore, USA). For cell apoptosis assay, cells were washed with D‐Hanks solution and suspended in 1 × binding buffer solution. After staining with Annexin V‐APC reagent (eBioscience, USA) for 10 min, the cell suspension was subjected to apoptosis detection. For cell cycle analysis, cells were washed with D‐Hanks solution and fixed in 75% ethanol for 1 h. The ethanol was then removed by centrifugation and cells were suspended in D‐Hanks solution containing propidium iodide (Sigma‐Aldrich) and RNase A reagent (Fermentas, Canada) before cell cycle detection.

### Colony formation assay

CRC cells in logarithmic phase were seeded into a 6‐well plate, with 1000 and 1500 cells per well for SW480 and SW620, respectively. After incubation for 14 days, cells were fixed with 4% paraformaldehyde for 30 min and stained with GIEMSA reagent (Dingguo Biotechnology, China) for 20 min. After washes with double distilled water, the colony number was counted under microscope.

### Xenograft models

A total of 12 female BALB/c nude mice (4–5 weeks old, 16.3 ± 2.5 g) were purchased from Shanghai experimental animal center of Chinese Academy of Sciences. The animal experimental protocol was approved by the Animal Care and Use Committee of Shanghai Tenth People's Hospital, Tongji University School of Medicine. Briefly, 5 × 10^6^ SW620 cells suspending in serum‐free medium were subcutaneously injected into the left armpit of each mouse. Tumor length (a) and width (b) was measured every 3 days using a caliper and the volume was calculated as follows: volume = *ab* ^2^/2. After 20 days, the mice were sacrificed and tumors were harvested.

### Microarray and ingenuity pathway analysis

The microarray analysis was performed on PrimeView^™^ Human Gene Expression Array (Affymetrix, USA) according to our previous description 13. In brief, total RNA was extracted from CRC cells transfected with shRNA and its negative control. The RNA sample was transferred onto Nanodrop 2000 (Thremo Fisher Scientific, USA) and 2100 Bioanalyzer (Aglient, USA) for quality control. The samples were subjected to In vitro transcription (IVT) assay using GeneChip 3′IVT Express Kit (Affymetrix, USA) and the obtained cRNA was labeled. Finally, the GeneChips were hybridized, washed and scanned according to the manufacturer's instructions. Quality control of microarray data was performed using signal histogram, relative signal box plot and Pearson's correlation analysis.

The microarray data were then processed by Ingenuity Pathway Analysis (IPA) online (www.ingenuity.com). The inclusion criteria for identifying significantly expressed genes were as follows: (1) *P* < 0.05; (2) fold change value > 1.5. IPA includes canonical pathway, disease, and function analysis. The potential eligible genes were further validated by qRT‐PCR and western blot.

### Statistical analysis

The data were present as mean ± standard deviation and analyzed in SPSS 22.0 statistical software (SPSS, USA). The in vitro/vivo cellular assays were analyzed using Student's *t* test. The correlations between NDC80 expression and clinicopathological parameters were analyzed using chi‐square test. The survival curves were constructed using Kaplan–Meier model and analyzed using the log‐rank test. Significant prognostic factors and their independence were evaluated using the univariate and multivariate analysis based on the Cox proportional hazards. A *P* value less than 0.05 indicates statistically significant.

## Results

### Expression and clinical correlations of NDC80 in CRC patients

**Figure 1:**
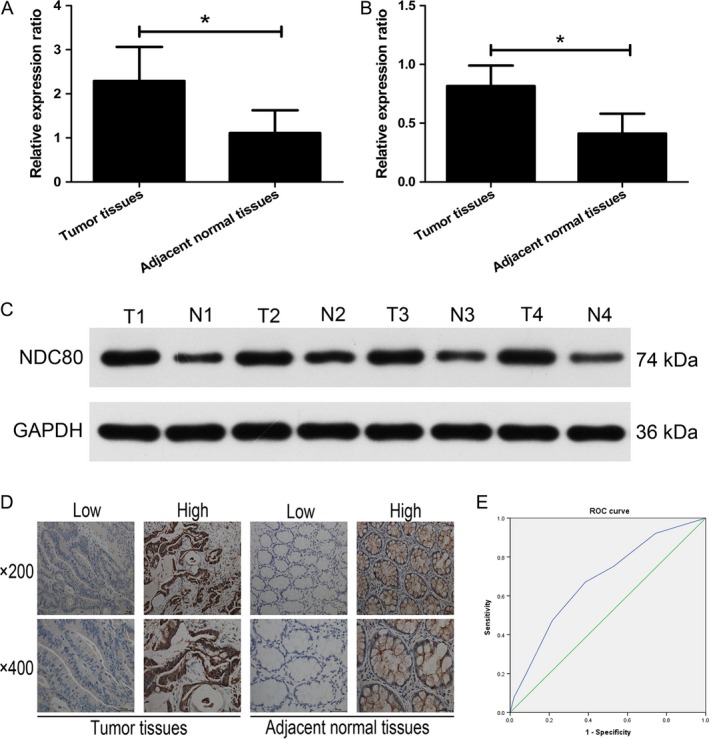
Expression of NDC80 in CRC and adjacent normal tissues. (A) The mRNA expression of NDC80 is significantly higher in CRC tissues than that in adjacent normal tissues (2.2915 ± 0.7726 vs. 1.1101 ± 0.5185, *n* = 25, *P* < 0.001). (B) The protein expression of NDC80 is significantly higher in CRC tissues than that in adjacent normal tissues (0.8172 ± 0.1739 vs. 0.4116 ± 0.1687, *n* = 25, *P* < 0.001). (C) Representative protein bands of western blot. (D) Representative images of immunohistochemical staining. (E) The ROC curve analysis determined the cut‐off value of staining scores is 3.5.*P < 0.05.

First, the mRNA expression of NDC80 in CRC and adjacent normal tissues was detected by qRT‐PCR. As shown in Figure 1A, the mRNA expression of NDC80 is significantly higher in CRC tissues than that in adjacent normal tissues (2.2915 ± 0.7726 vs. 1.1101 ± 0.5185, *n* = 25, *P* < 0.001). The western blot was then performed to demonstrate that its protein expression remains to be significantly higher in CRC tissues than that in adjacent normal tissues (0.8172 ± 0.1739 vs. 0.4116 ± 0.1687, *n* = 25, *P* < 0.001, Fig. 1B and C). For further investigating the clinical correlation of NDC80 in CRC patients, immunohistochemical staining combined with semiquantitative evaluation was employed and the representative results were demonstrated in Figure 1D. The ROC curve analysis suggests the cut‐off value of staining scores is 3.5 (Fig. 1E), and therefore the entire patient cohort was classified into high expression group (*n* = 108) and low expression group (*n* = 116) based on it.

The correlations of NDC80 expression with clinical features were summarized in Table 1. NDC80 expression is significantly correlated with tumor invasion, lymph node and distant metastasis (*P* = 0.041, *P* = 0.013 and *P* = 0.015), but not with other features including gender, age, tumor location, colon tumor location, tumor size, and tumor differentiation (*P* = 0.678, *P* = 0.520, *P* = 0.733, *P* = 0.381, *P* = 0.602, and *P* = 0.264).

**Table 1:** Correlations between NDC80 expression and clinicopathological characteristics in CRC patients

Characteristics	Total	NDC80 expression		*P* value
		Low	High	
Gender				
Female	109	58	51	0.678
Male	115	58	57	
Age				
≤65 years	68	33	35	0.520
>65 years	156	83	73	
Tumor location				
Rectal	73	39	34	0.733
Colon	151	77	74	
Colon tumor location				
Left side	83	45	38	0.381
Right side	68	32	36	
Tumor size				
≤5 cm	149	79	70	0.602
>5 cm	75	37	38	
Tumor differentiation				
Poor	41	18	23	0.264
Well/moderate	183	98	85	
Tumor invasion				
*T*_1_−*T*_2_	46	30	16	0.041
*T*_3_−*T*_4_	178	86	92	
Lymph node metastasis				
N0	99	56	43	0.013
N1	70	41	29	
N2	55	19	36	
Distant metastasis				
Absent	202	110	92	0.015
Present	22	6	16	

### Prognostic significance of NDC80 in CRC patients

**Figure 2:**
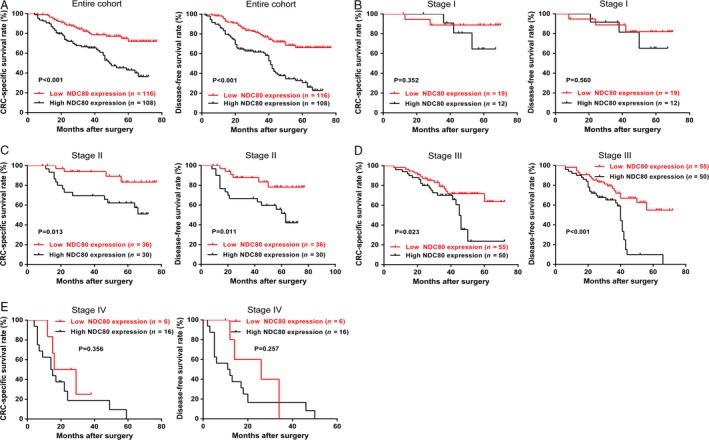
Prognostic significance of NDC80 in CRC patients. (A) In the entire cohort, patients with high NDC80 expression had a worse CRC‐specific survival (CSS) and disease‐free survival (DFS) than those with low NDC80 expression. (B) No significant association was found between NDC80 expression and CSS/DFS in stage I patients. (C) High NDC80 expression is significantly associated with worse CSS and DFS in stage II patients. (D) High NDC80 expression is significantly associated with worse CSS and DFS in stage III patients. (E) No significant association was found between NDC80 expression and CSS/DFS in stage IV patients.

The impact of NDC80 on patient prognosis was assessed using Kaplan–Meier survival curves. As shown in Figure 2A, for the entire cohort, high NDC80 expression is associated with worse CSS and DFS than low NDC80 expression (*P *<* *0.001 and *P *<* *0.001). As shown in Table 2, the following univariate analysis indicates NDC80 expression, tumor invasion, lymph node metastasis, and distant metastasis were significant prognostic factors for CSS (*P* < 0.001, *P* = 0.001, *P* < 0.001, *P* < 0.001), whereas NDC80 expression, tumor differentiation, tumor invasion, lymph node metastasis, and distant metastasis were for DFS (*P* < 0.001, *P* = 0.018, *P* < 0.001, *P* < 0.001, *P* < 0.001). The multivariate analysis further reveals NDC80 expression, tumor differentiation, tumor invasion, lymph node metastasis, and distant metastasis were independent significant prognostic factors for both CSS and DFS (CSS: *P* = 0.009, *P* = 0.016, *P* = 0.024, *P* = 0.021, *P* < 0.001; DFS: *P* < 0.001, *P* = 0.004, *P* = 0.032, *P* = 0.001, *P* < 0.001, Table 3). Finally subgroup analysis was performed to investigate whether NDC80 expression is helpful for predicting clinical outcome of patients within the same TNM stage. As shown in Figure 2B–E, NDC80 expression could stratify the CSS and DFS in stage II and III patients (stage II: *P* = 0.013 and *P* = 0.011; stage III: *P* = 0.023 and *P* < 0.001), but failed in stage I and IV patients (stage I: *P* = 0.352 and *P* = 0.560; stage IV: *P* = 0.356 and *P* = 0.257).

**Table 2:** Univariate analysis for prognostic factors affecting CRC‐specific survival and disease‐free survival

Variables	CRC‐specific survival			Disease‐free survival		
	HR	95% CI	*P* value	HR	95% CI	*P* value
Age	1.273	0.757–2.141	0.362	0.913	0.593–1.405	0.677
Gender	1.227	0.781–1.929	0.375	1.185	0.794–1.767	0.407
Tumor location	1.266	0.766–2.094	0.357	0.969	0.634–1.479	0.882
Tumor size	1.014	0.631–1.629	0.953	1.284	0.854–1.930	0.230
Tumor differentiation	1.630	0.947–2.806	0.078	1.763	1.101–2.824	0.018
Tumor invasion	4.873	1.965–12.083	0.001	3.472	1.746–6.905	<0.001
Lymph node metastasis	1.949	1.474–2.578	<0.001	1.982	1.547–2.540	<0.001
Distant metastasis	7.315	4.228–12.655	<0.001	6.678	3.973–11.224	<0.001
NDC80 expression	2.603	1.612–4.204	<0.001	2.860	1.864–4.387	<0.001

**Table 3:** Multivariate analysis for prognostic factors affecting CRC‐specific survival and disease‐free survival

Variables	CRC‐specific survival			Disease‐free survival		
	HR	95% CI	*P* value	HR	95% CI	*P* value
Age	1.570	0.896–2.752	0.115	1.119	0.703–1.781	0.635
Gender	0.967	0.604–1.548	0.888	0.964	0.634–1.465	0.864
Tumor location	1.038	0.612–1.761	0.891	0.800	0.512–1.251	0.329
Tumor size	1.033	0.633–1.684	0.897	1.324	0.868–2.020	0.193
Tumor differentiation	2.047	1.142–3.671	0.016	2.118	1.270–3.530	0.004
Tumor invasion	2.987	1.151–7.752	0.024	2.240	1.073–4.677	0.032
Lymph node metastasis	1.431	1.055–1.942	0.021	1.555	1.192–2.029	0.001
Distant metastasis	4.467	2.478–8.052	<0.001	3.904	2.261–6.740	<0.001
NDC80 expression	1.936	1.181–3.174	0.009	2.218	1.431–3.440	<0.001

### Silencing NDC80 expression inhibits the growth of CRC cells in vitro

**Figure 3:**
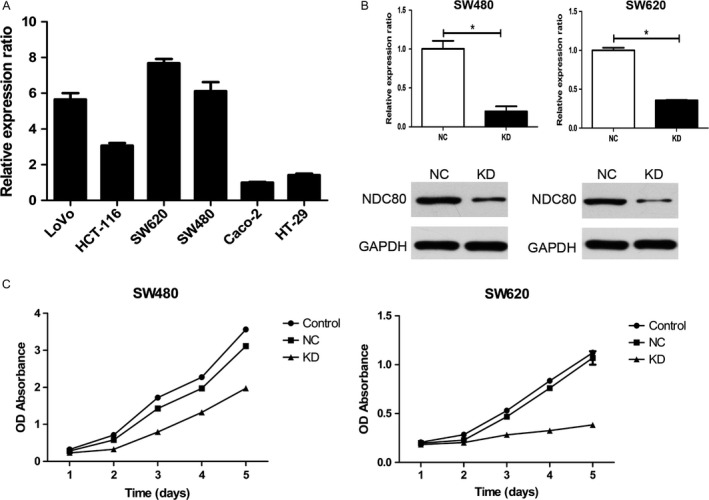
Silencing NDC80 expression inhibits the proliferative ability of CRC cells. (A) The qRT‐PCR demonstrated the mRNA expression of NDC80 is highest in SW620 and SW480 CRC cell lines. (B) Both the qRT‐PCR and western blot confirmed the expression of NDC80 was decreased in SW480 (left) and SW620 (right) CRC cells after lentivirus transfection. (C) Silencing NDC80 expression inhibits the proliferation of SW480 (left) and SW620 (right) CRC cells. *P < 0.05.

First, qRT‐PCR was utilized to detect the mRNA expression of NDC80 in various CRC cell lines and the result suggests it appears to be highest in SW620 and SW480 cells (Fig. 3A). Therefore, both the cell lines were selected for the following cellular assays. Then, shRNA was used to downregulate NDC80 expression in CRC cells and its efficacy was examined. As shown in Figure 3B, for SW480 and SW620 cells, both qRT‐PCR and western blot demonstrated NDC80 expression was dramatically decreased in knockdown (KD) group as compared with that in negative control (NC) group (PCR: all *P* < 0.001). The following CCK‐8 assay indicated the proliferative abilities of both SW620 and SW480 cells were obviously inhibited after silencing NDC80 expression (all *P* < 0.001, Fig. 3C).

### Silencing NDC80 expression increases apoptosis rate and arrests cell cycle in CRC cells

**Figure 4:**
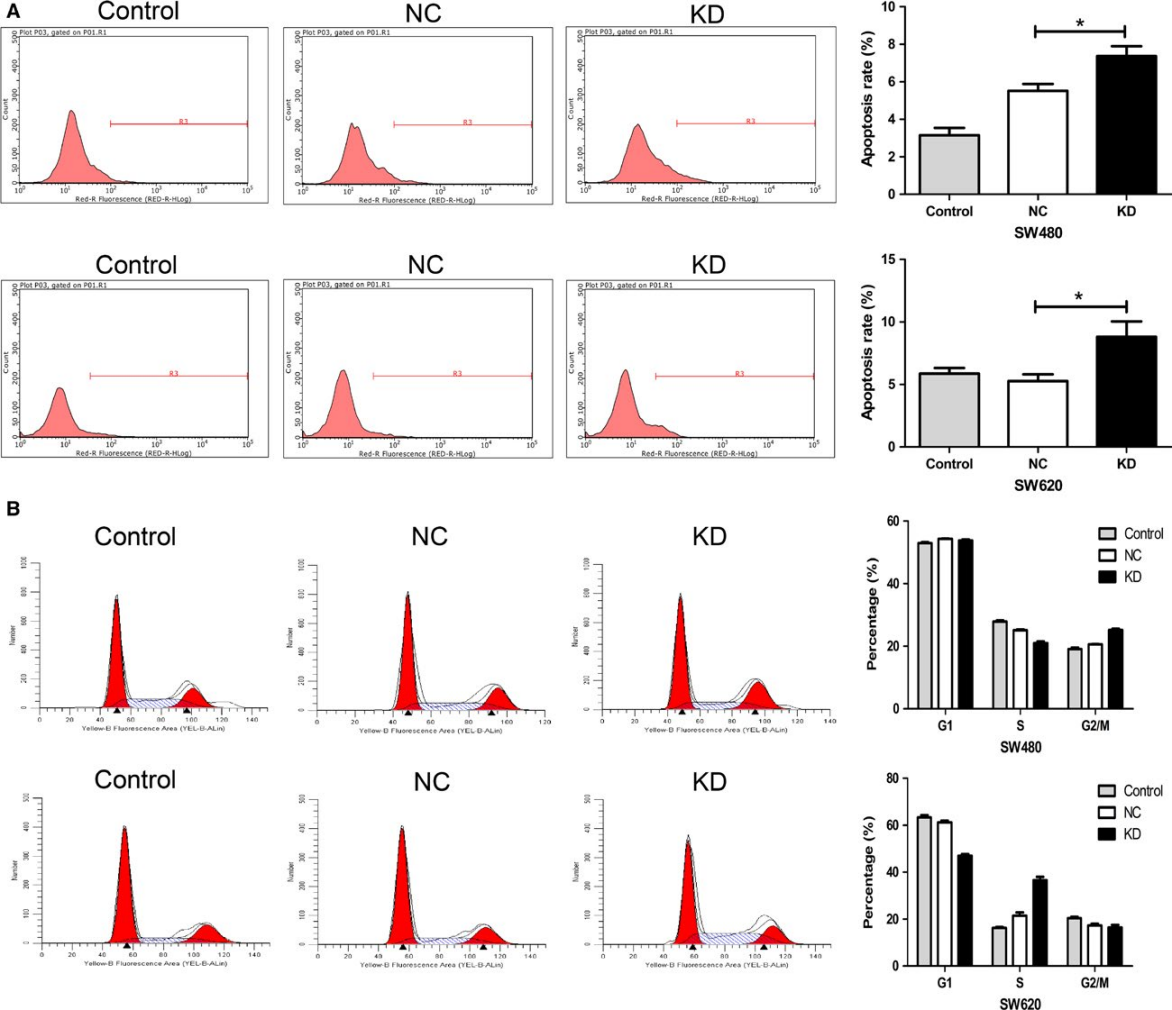
Silencing NDC80 expression induces the apoptosis and arrests the cell cycle of CRC cells. (A) Silencing NDC80 expression increases the apoptosis rate of SW480 (upper) and SW620 (lower) cells. (B) Silencing NDC80 expression arrests cell cycle progression in G2/M phase in SW480 cells (upper) and in S phase in SW620 cells (lower), respectively.*P < 0.05.

As shown in Figure 4A, for both SW480 and SW620 cells, the apoptosis rate of KD group is significantly higher than that of NC group (*P* = 0.0077 and *P* = 0.0105). In cell cycle assay, silencing NDC80 expression decreases the S phase fraction and increases the G2/M phase fraction in SW480 cells, whereas it decreases the G1 phase fraction and increases the S phase fraction in SW620 cells (all *P* < 0.05, Fig. 4B). Nevertheless, both the results collectively suggest downregulating NDC80 induces cell cycle arrest of CRC cells.

### Silencing NDC80 expression inhibits tumorigenic potential of CRC cells in vitro and in vivo

**Figure 5:**
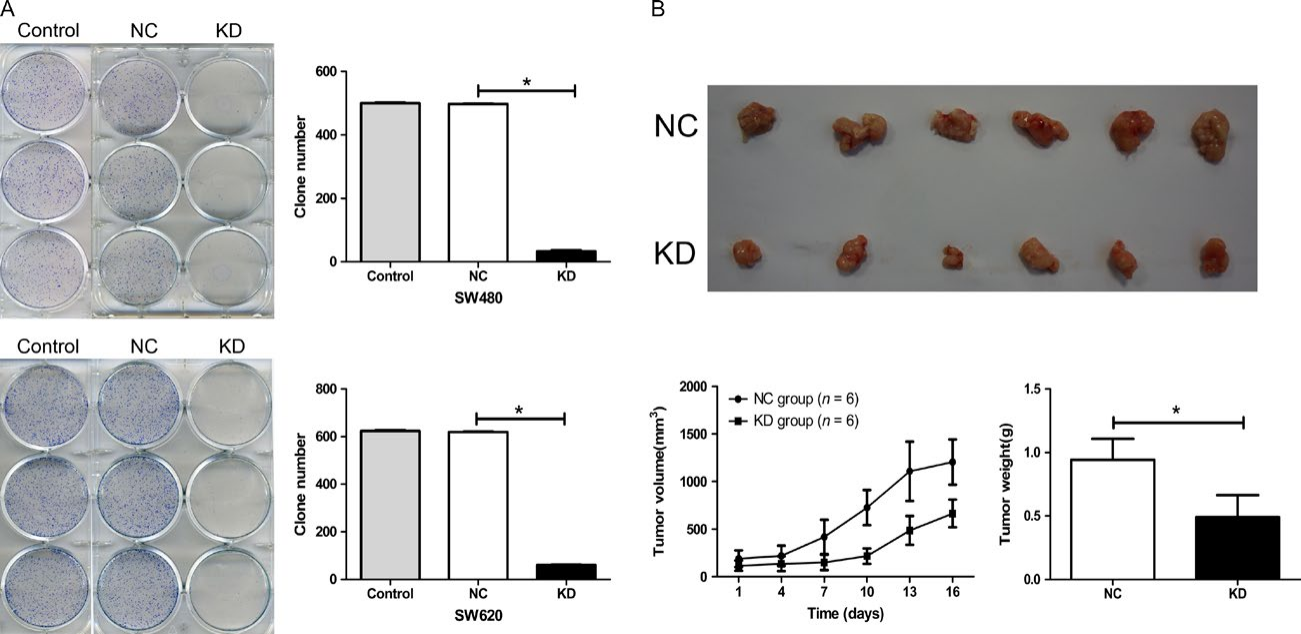
Silencing NDC80 expression inhibits the tumorigenic potential of CRC cells in vitro and in vivo. (A) Silencing NDC80 expression decreases the clone number of SW480 (upper) and SW620 cells (lower). (B) Upper: Silencing NDC80 expression reduces the sizes of the harvested CRC xenografts; Lower: Silencing NDC80 expression inhibits the growth (left) and weight (right) of CRC xenografts.*P < 0.05.

The tumorigenic potential of CRC cells in vitro was assessed by colony formation assay. For both SW480 and SW620 cells, the clones of KD group are significantly fewer than that of NC group (all *P* < 0.001, Fig. 5A). Then, a xenograft model was established to assess the impact of NDC80 on the tumorigenic potential of SW620 cells in vivo and the harvested tumors are shown in Figure 5B. The tumor volumes and weights of KD groups are both dramatically smaller than those of NC groups (*P* < 0.001 and *P* = 0.001). Taken together, these findings suggest NDC80 is crucial for tumorigenic potential of CRC cells both in vitro and in vivo.

### NDC80 promotes CRC progression through downregulating DUSP5 and FOXO1

**Figure 6:**
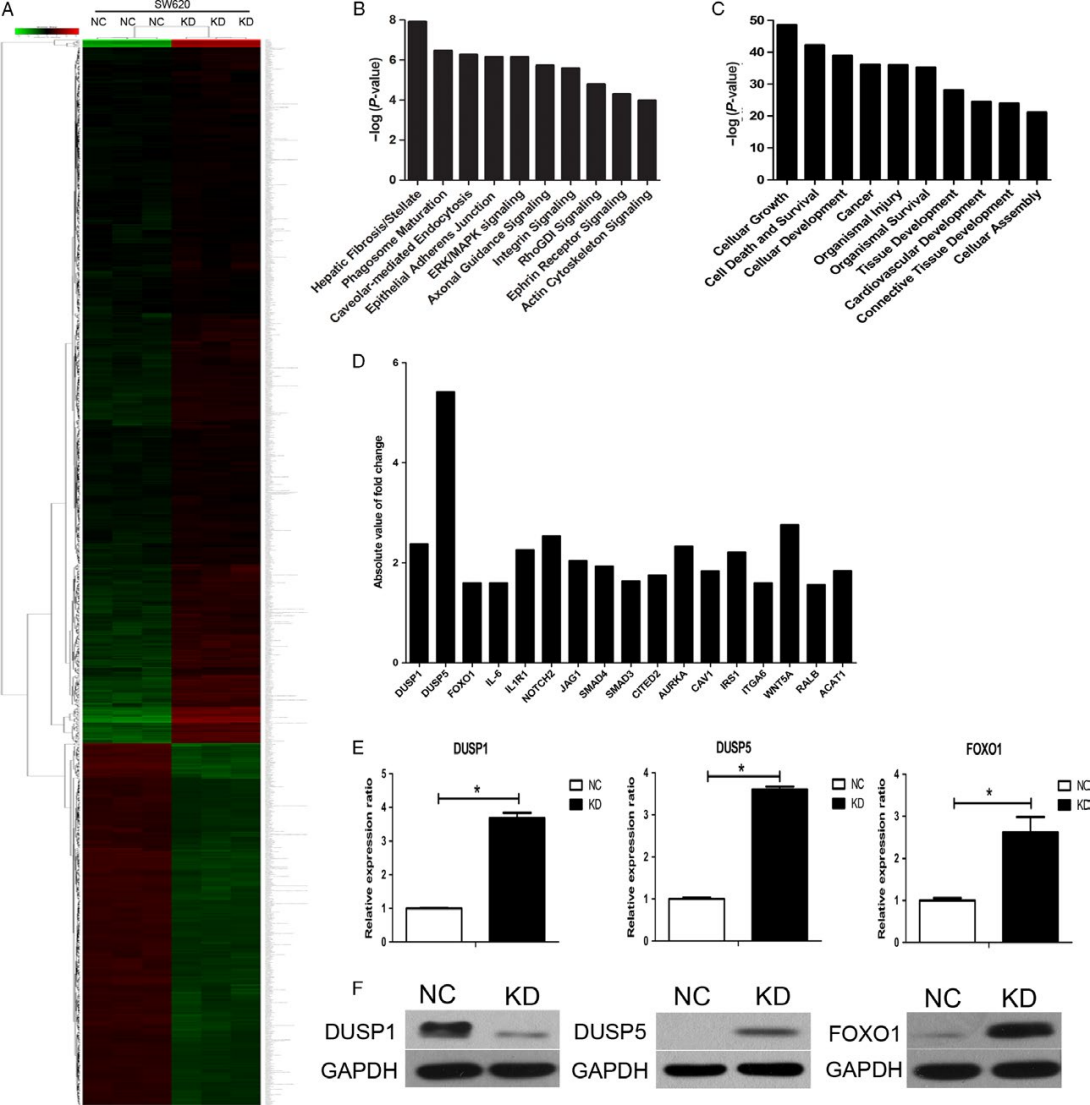
NDC80 promotes CRC progression partly by downregulating DUSP5 and FOXO1. (A) Heat map depicting significantly upregulated/downregulated genes after silencing NDC80 expression in SW620 cells. (B) Canonical pathway analysis. (C) Disease and function analysis. (D) Absolute fold change values of selected genes. (E) The qRT‐PCR validates the mRNA expression of representative affected genes after silencing NDC80 expression in SW620 cells. (F) Western blot validates the protein expression of DUSP1, DUSP5, and FOXO1 after silencing NDC80 expression in SW620 cells.*P < 0.05.

The microarray analysis was utilized to speculate the potential oncogenic mechanisms regulated by NDC80 in CRC development. In general, a total of 879 and 489 genes are significantly upregulated and downregulated, respectively, after silencing NDC80 expression in SW620 cells (Fig. 6A). The canonical pathway analysis indicates these genes are enriched in some cancer‐related signals such as Extracellular regulated protein kinases/Mitogen‐activating protein kinase (ERK/MAPK) signaling and integrin signaling (Fig. 6B). The disease and function analysis also indicates these genes mainly participate in some cancer‐related biological processes such as cellular growth, cell death, and survival (Fig. 6C). To further clarify the involved mechanism, 17 representative significantly expressed genes were selected based on their potential oncogenic roles and their fold change values were shown in Figure 6D. Then, qRT‐PCR was performed to validate their expression in NDC80 silencing SW620 cells and the results are shown in Figure 6E and Figure S1. Unfortunately only three genes, dual specificity phosphatase 1 (DUSP1), dual specificity phosphatase 5 (DUSP5), and forkhead box O1 (FOXO1), remain significantly expressed and have the same expression pattern with the microarray data. Then, we performed western blot to validate their protein expression in CRC cells. After silencing NDC80 in SW620 cells, we found the protein expression of DUSP5 and FOXO1 was significantly increased, whereas the opposite was for that of DUSP1, which is somewhat inconsistent with PCR detection (Fig. 6F). Therefore, considering these findings, we preliminarily speculate NDC80 promotes CRC progression partly through downregulating DUSP5 and FOXO1.

## Discussion

In this study, we compared the expression of NDC80 between tumor and adjacent normal tissues, and identified it as a potential prognostic biomarker for CRC patients. In addition, functional assays proved NDC80 enhanced the malignant characteristics of CRC cells in vitro and in vivo. Finally, microarray analysis preliminarily suggested its involved oncogenic molecular mechanisms during CRC development. In this regard, our findings are supportive of a recent review highlighting the crucial role of NDC80 in promoting tumor initiation and development 14.

Using qRT‐PCR and western blot, we firstly quantified NDC80 expression in clinical specimens and found it was dramatically higher in CRC tissues as compared with that in adjacent normal tissues. This observation is consistent with previous studies that demonstrated its abnormal overexpression in other solid tumors such as prostate, pancreas, and liver cancer 9, 10, 15. For further identifying its potential as a clinical biomarker, we analyzed its expression in a retrospective cohort enrolling 224 CRC patients. The results suggested NDC80 expression is correlated with tumor invasion, lymph node, and distant metastasis, implying its involvement in CRC progression. The following survival analysis showed CRC patients with high NDC80 expression have a significantly worse outcome than those with low NDC80 expression. In addition, the multivariate analysis revealed its independence as an unfavorable prognostic factor affecting CSS and DFS. These evidences strongly supported that NDC80 is a potential biomarker for predicting disease progression and patient prognosis. Similarly, our previous study has also validated it as an independent unfavorable prognostic predictor for osteosarcoma patients, partly supporting our present results 11. Using bioinformatic analysis, NDC80 was identified as a candidate prognostic biomarker for breast cancer survivors, which may be beneficial for evaluating adjuvant therapy response and cancer recurrence risk 16. Then, we performed subgroup analysis to investigate the prognostic significance of NDC80 in CRC patients within the same TNM stage and found its expression could stratify the clinical outcome in stage II and III patients but failed in stage I and IV patients. This result, on the one hand, suggested evaluating NDC80 expression in primary tumors may contribute to a more precise prognostic classification for stage II and III patients. On the other hand, we are unable to make a definite conclusion for its prognostic significance in stage I and IV patients because our sample size is relatively limited. To tackle this issue, more validations based on sufficient clinical resources are needed.

Considering that NDC80 overexpression is correlated with advanced tumor stage and unfavorable prognosis, we then performed functional assays to investigate its specific biological role in CRC cells. We found knockdown of NDC80 significantly inhibits cell proliferation, increases apoptosis rate and arrests cell cycle in both SW620 and SW480 CRC cells. Furthermore, through clone formation assay and xenograft models, we proved that NDC80 is crucial for the tumorigenic potential of CRC cells in vitro and in vivo. These findings are in accordance with a recent work that demonstrated NDC80 promotes the growth of gastric cancer cells in vitro and in vivo 17. Liu et al. also found knockdown of NDC80 inhibits the proliferation of gliomas cells and downregulates Ki‐67 expression 18. The related mechanism investigation suggested inactivation of pRb signaling pathway increases NDC80 expression and leads to the uncontrolled cell cycle progression of cancer cells 19. Taken together, these evidences strongly support that NDC80 plays an important role in CRC development. Finally, it should be mentioned that TAI‐95, a NDC80 inhibitor, has been proved to be superior in inhibiting the in vitro growth of primary liver cancer cells as compared with some current targeted agents such as sorafenib 20. Through its N‐terminus‐modification, NDC80 can be even utilized to inhibit proliferation and induces apoptosis of cervical cancer cells 21. Both the recent achievements suggest that targeting NDC80 may be a promising individualized therapeutical strategy for CRC patients. However, establishing standardized detection for NDC80 expression and designing safe agents to precisely target NDC80 in CRC cells still require massive efforts in future.

Finally, microarray analysis preliminarily revealed some oncogenic mechanisms regulated by NDC80. First, the disease and function analysis suggested differentially expressed genes affected by NDC80 knockdown mainly participate in biological processes such as cellular growth, cell death, and survival, which is in accordance with our observations in functional assays. Second, the canonical pathway analysis indicates these genes were also partly enriched in ERK/MAPK and integrin signaling pathways, both of which are currently known as key driving factors in CRC development 22, 23. Third, we focused on some representative differentially expressed genes and validated their expression in CRC cells. As a result, we found knockdown of NDC80 significantly increases the expression of DUSP5 and FOXO1 both at mRNA and protein level. DUSP5 is a well‐established tumor suppressor exerting its anticancer effects through inactivating MAP signaling pathway 24. Our previous study has identified its expression is negatively correlated with epithelial‐mesenchymal transition phenotype and serves as a favorable prognostic indicator for advanced CRC patients 25. Similar with DUSP5, FOXO1 is recently found to inactivate MAPK signaling pathway through impeding ERK1/2 phosphorylation, leading enhanced chemosensitivity of cancer cells 26. Accumulating evidences have suggested it acts as a tumor suppressor in various human malignancies such as gastric, endometrial and bladder cancer 27, 28, 29. In CRC, FOXO1 is activated by NLR family CARD domain containing 3 to exert its inhibitory role in cellular proliferation 30. Therefore, considering our preliminary findings and previous related evidences, we speculate NDC80 promotes CRC progression partly by downregulating DUSP5 and FOXO1.

Despite our novel findings in this study, there two potential deficiencies that merit attention in our following work. The one is that we failed to prove the significant correlation of NDC80 with patient prognosis in stage I/IV CRC. This discrepant result in subgroup analysis may be largely attributed to the limited tissue samples in our study and therefore more related validations should be performed based on sufficient clinical resources in the future. The other one is that we failed to completely clarify the oncogenic molecular mechanisms regulated by NDC80 in CRC cells. In addition, the specific signaling pathways regulated by DUSP5 or FOXO1 in CRC are also unclear. To tackle this issue, more experiments in vitro and in vivo are needed in our following investigation.

In summary, our results indicate that high NDC80 expression is correlated with advanced tumor stage and unfavorable prognosis in CRC patients. In addition, we also find NDC80 drives malignant progression of CRC cells partly by inactivating DUSP5 and FOXO1. Taken together, these evidences suggest NDC80 is not only a promising clinical biomarker for patient management, but also a potential therapeutical target for CRC diagnosis and treatment.

## Conflict of Interest

The authors declare that they have no conflict of interest.

## Supporting information


**Figure S1.** The qRT‐PCR validates the mRNA expression of significantly expressed genes after silencing NDC80 expression in SW620 cells. (A) Interleukin‐6 (IL‐6). (B) NOTCH2. (C) Jagged 1 (JAG1). (D) SMAD family member 4 (SMAD4). (E) Cbp/p300 interacting transactivator with Glu/Asp rich carboxy‐terminal domain 2 (CITED2). (F) SMAD family member 3 (SMAD3). (G) Aurora kinase A (AURKA). (H) Caveolin 1 (CAV1). (I) Insulin receptor substrate 1 (IRS1). (J) Integrin subunit alpha 6 (ITGA6). (K) Wnt family member 5A (WNT5A). (L) RAS like proto‐oncogene B (RALB). (M) Interleukin 1 receptor type 1 (IL1R1). (N) Acetyl‐CoA acetyl transferase 1 (ACAT1).

**Table S1.** Primer sequences for the quantitative polymerase chain reaction.
