# Supplementary figures and images for: Nuclear division cycle 80 promotes malignant progression and predicts clinical outcome in colorectal cancer
Source: Cancer Med. 2018 Jan 17;7(2):420–32. doi: 10.1002/cam4.1284 (PMC5806104; doi:10.1002/cam4.1284)

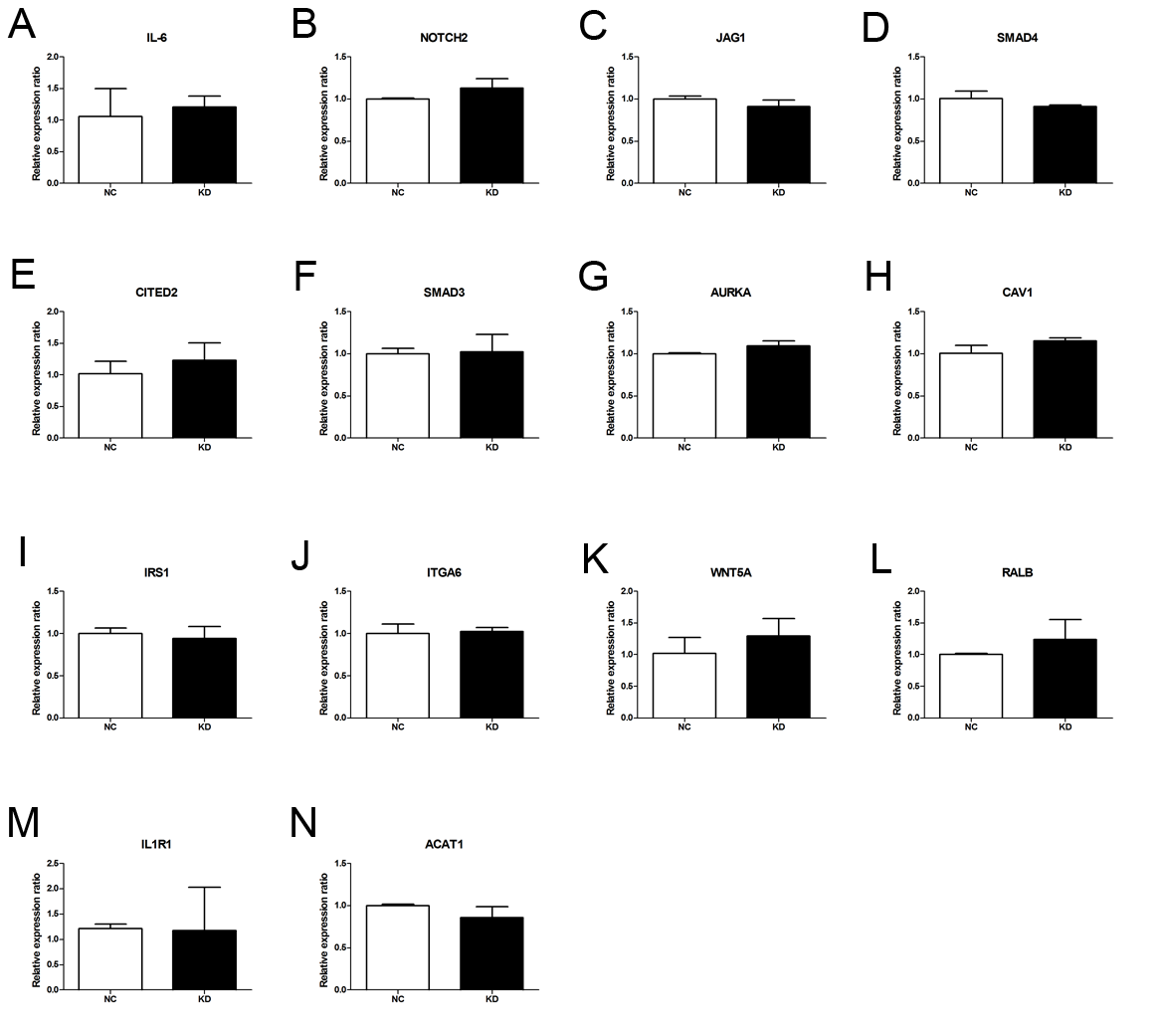

Supplement: Supplementary file 1 — Figure S1. The qRT‐PCR validates the mRNA expression of significantly expressed genes after silencing NDC80 expression in SW620 cells. (A) Interleukin‐6 (IL‐6). (B) NOTCH2. (C) Jagged 1 (JAG1). (D) SMAD family member 4 (SMAD4). (E) Cbp/p300 interacting transactivator with Glu/Asp rich carboxy‐terminal domain 2 (CITED2). (F) SMAD family member 3 (SMAD3). (G) Aurora kinase A (AURKA). (H) Caveolin 1 (CAV1). (I) Insulin receptor substrate 1 (IRS1). (J) Integrin subunit alpha 6 (ITGA6). (K) Wnt family member 5A (WNT5A). (L) RAS like proto‐oncogene B (RALB). (M) Interleukin 1 receptor type 1 (IL1R1). (N) Acetyl‐CoA acetyl transferase 1 (ACAT1). [file CAM4-7-420-s001.tif]
