# Supplementary material for: Nuclear division cycle 80 promotes malignant progression and predicts clinical outcome in colorectal cancer
Source: Cancer Med. 2018 Jan 17;7(2):420–32. doi: 10.1002/cam4.1284 (PMC5806104; doi:10.1002/cam4.1284)
Supplement: Supplementary file 2 — Table S1. Primer sequences for the quantitative polymerase chain reaction. [file CAM4-7-420-s002.docx]

**Supplementary Table.** Primer sequences for the quantitative polymerase chain reaction.

| Gene | Orientation | Sequence (5'‑3') |
| --- | --- | --- |
| NDC80 | Forward | CCTCTCCATGCAGGAGTTAAGA |
|  | Reverse | GGTCTCGGGTCCTTGATTTTCT |
| DUSP1 | Forward | GCAGTACCCCACTCTACGATC |
|  | Reverse | TTGAACCAGGAGCTGATGTCT |
| DUSP5 | Forward | TCCTCACCTCGCTACTCG |
|  | Reverse | ACATCCACGCAACACTCAG |
| FOXO1 | Forward | GAGTGGATGGTCAAGAGCGT |
|  | Reverse | TTCCTTCATTCTGCACACGA |
| IL6 | Forward | CAAATTCGGTACATCCTCG |
|  | Reverse | CTCTGGCTTGTTCCTCACTA |
| NOTCH2 | Forward | AGGGTAGTAGGAGGAAGAAGTC |
|  | Reverse | TGGAGAGGATGTGGTGTCG |
| JAG1 | Forward | ATGGTTATCGCTGTATCTGTCC |
|  | Reverse | GTCACTGGCACGGTTGTAG |
| SMAD4 | Forward | ACGAACGAGTTGTATCACCTGG |
|  | Reverse | TGCACGATTACTTGGTGGATG |
| CITED2 | Forward | GACAAACCAGCACTTCCG |
|  | Reverse | CCAAACCCATTTCTATCAC |
| SMAD3 | Forward | CCTTTCAGGTAACCGTCTT |
|  | Reverse | TTTAGCCCATCATCTCCC |
| AURKA | Forward | GCCCTGTCTTACTGTCATTCG |
|  | Reverse | AGGTCTCTTGGTATGTGTTTGC |
| CAV1 | Forward | CTGAGCGAGAAGCAAGTG |
|  | Reverse | AGAGAGAATGGCGAAGTAAATG |
| IRS1 | Forward | ACAAACGCTTCTTCGTACTGC |
|  | Reverse | AGTCAGCCCGCTTGTTGATG |
| ITGA6 | Forward | GGCGGTGTTATGTCCTGAGTC |
|  | Reverse | AATCGCCCATCACAAAAGCTC |
| WNT5A | Forward | TCGACTATGGCTACCGCTTTG |
|  | Reverse | CACTCTCGTAGGAGCCCTTG |
| RALB | Forward | TCATCAGGAAAGGAGCACT |
|  | Reverse | GAGGGGATACAGGATTGTT |
| IL1R1 | Forward | GGCCAGTTGAGTGACATTGCT |
|  | Reverse | TGTGATGAGGGTACTCCTTCTTT |
| ACAT1 | Forward | AAGGCAGGCAGTATTGGGTG |
|  | Reverse | ACATCAGTTAGCCCGTCTTTTAC |
| GAPDH | Forward | TGACTTCAACAGCGACACCCA |
|  | Reverse | CACCCTGTTGCTGTAGCCAAA |

Abbreviation：NDC80: Nuclear division cycle 80; DUSP1: Dual specificity phosphatase 1; DUSP5: Dual specificity phosphatase 5; FOXO1: Forkhead box O1; PDGFA: Platelet derived growth factor A; SGK1: Serum/glucocorticoid regulated kinase 1; GNG4: G protein subunit gamma 4; IL-6: Interleukin-6; JAG1: Jagged 1; SMAD4: SMAD family member 4; CITED2: Cbp/p300 interacting transactivator with Glu/Asp rich carboxy-terminal domain 2; SMAD3: SMAD family member 3; AURKA: Aurora kinase A; CAV1: Caveolin 1; IRS1: Insulin receptor substrate 1; ITGA6: Integrin subunit alpha 6; WNT5A: Wnt family member 5A; RALB: RAS like proto-oncogene B; IL1R1: Interleukin 1 receptor type 1; ACAT1: Acetyl-CoA acetyl transferase 1; GAPDH: Glyceraldehyde-3-phosphate dehydrogenase
